# Inter-row navigation line detection for cotton with broken rows

**DOI:** 10.1186/s13007-022-00913-y

**Published:** 2022-07-02

**Authors:** Xihuizi Liang, Bingqi Chen, Chaojie Wei, Xiongchu Zhang

**Affiliations:** 1 Institute of intelligent manufacturing, Suzhou Chien-Shiung Institute of Technology, Suzhou, Jiangsu China; 2 College of Engineering, China Agricultural University, Beijing, China

**Keywords:** Crop rows detection, Machine vision, Autonomous navigation, Intra-row line

## Abstract

**Background:**

The application of autopilot technology is conductive to achieving path planning navigation and liberating labor productivity. In addition, the self-driving vehicles can drive according to the growth state of crops to ensure the accuracy of spraying and pesticide effect. Navigation line detection is the core technology of self-driving technology, which plays a more important role in the development of Chinese intelligent agriculture. The general algorithms for seedling line extraction in the agricultural fields are for large seedling crops. At present, scholars focus more on how to reduce the impact of crop row adhesion on extraction of crop rows. However, for seedling crops, especially double-row sown seedling crops, the navigation lines cannot be extracted very effectively due to the lack of plants or the interference of rut marks caused by wheel pressure on seedlings. To solve these problems, this paper proposed an algorithm that combined edge detection and OTSU to determine the seedling column contours of two narrow rows for cotton crops sown in wide and narrow rows. Furthermore, the least squares were used to fit the navigation line where the gap between two narrow rows of cotton was located, which could be well adapted to missing seedlings and rutted print interference.

**Results:**

The algorithm was developed using images of cotton at the seedling stage. Apart from that, the accuracy of route detection was tested under different lighting conditions and in maize and soybean at the seedling stage. According to the research results, the accuracy of the line of sight for seedling cotton was 99.2%, with an average processing time of 6.63 ms per frame; the accuracy of the line of sight for seedling corn was 98.1%, with an average processing time of 6.97 ms per frame; the accuracy of the line of sight for seedling soybean was 98.4%, with an average processing time of 6.72 ms per frame. In addition, the standard deviation of lateral deviation is 2 cm, and the standard deviation of heading deviation is 0.57 deg.

**Conclusion:**

The proposed rows detection algorithm could achieve state-of-the-art performance. Besides, this method could ensure the normal spraying speed by adapting to different shadow interference and the randomness of crop row growth. In terms of the applications, it could be used as a reference for the navigation line fitting of other growing crops in complex environments disturbed by shadow.

## Introduction

Farmland visual navigation is an important branch of intelligent agriculture. In the unstructured and random complex farmland environment, the visual navigation unmanned vehicle can not only monitor the walking path in real time according to the actual growth status of crops in the farmland, but also complete spraying operation efficiently without damaging the crops. With increasing attention from scholars at home and abroad, it has become a research hotspot of intelligent agriculture.

In the process of visual navigation of cotton spraying vehicle, the accurate acquisition of effective information in the image is the premise of whether the navigation vehicle can operate correctly [1–3]. Outdoor agricultural environments are characterized by uncontrolled and variable lighting conditions [4–8]. Shadows and over-intense or poor lighting are the major factors affecting the image quality [5–8]. In the seedling stage of cotton, some factors can affect the detection accuracy of crop rows in two main ways. First, cotton at the seedling stage has a shortage of crop plants due to the omission of seeding, lack of germination, being crushed by wheels, or insect/disease infestation. Second, cotton grows at different rates and in different heights, which can result in missing seedlings under the perspective projection of the image. When path detection is performed for straight-line work on seedling cotton, a straight line needs to be fitted to the gap between two narrow rows of cotton as the navigation line to be detected. There are two main factors causing two types of interference in the visual navigation images: (1) adhesions between two narrow rows of cotton far from the top end of the image; (2) significant gaps between plants in the same row of cotton. Thus, it is essential to effectively remove the row adhesions without making the gaps between the plants affect the segmentation of the cotton rows.

Least squares method, as one of the most common machine vision methods for identifying crop rows is aimed to deal with discontinuous lines, which has been used for real-time automatic guidance of agricultural vehicles [9]. Many scholars have studied the detection of navigation lines in seedling crops by different methods of finding feature points and fitting straight lines with least squares, and have achieved relatively good results [10–14], even for curved seedling columns, and have been able to detect navigation lines accurately [15]. However, there is a lack of research on visual navigation of farm fields with a small number of missing seedlings. Although many scholars have studied how to solve the problem of missing seeds by automatically replenishing seeds with a replenishing device at the time of sowing [16, 17], further research, including data evaluation and data mining, is needed to detect a few missing seedlings in crop rows for other causes of row breakage.

This paper presented an algorithm for detecting inter-row lines in seedling cotton. The inter-row sticking and broken rows resulted from small seedling leaves and gaps between two narrow rows are the complications that affect the visual navigation path detection in the six-row wide and narrow row cotton planting method (machine picked cotton). The detection of interlinear lines is investigated for the interference of broken lines in seedling cotton pictures, the method of navigating the detection of interplant lines in seedling cotton, and the optimization scheme of relevant parameters is analyzed according to the experimental environment. The remainder of this paper is organized as follows. “Introduction” section is the general introduction. “Material and methods**” **section describes materials and methods used for strategy in detail, which includes the segmentation and the classification of disease in the cotton leaf. “Experiment results” section shows the results obtained by employing the proposed method, and the discussion of the effect among proposed method and other plants. “Discussions” discussed the different parameters in this method. Lastly, the conclusions are summarized in “Conclusions” section.

## Material and methods

In this section, the principle of graph-cutting for the segmentation of cotton rows as well as backgrounds was reformulated for cotton at the bud stage. In addition, a new graph-cutting-OTSU method of image segmentation was proposed to improve detection accuracy and robustness. What’s more, an iterative least-squares method was put forward to accommodate the inhomogeneity and randomness of cotton growth.

### Image acquisition

The test video was collected in the field in Xinjiang Agricultural Division 7 cotton at 44°25′27.61″ N, 84°57′27.15″ E, 464 m above sea level, in an area with aridity and low rainfall, annual sunshine hours of 2721–2818 h, annual precipitation of 125.0–207.7 mm, and an average wind speed of 1.5 m/s, belonging to a typical temperate continental climate. Besides, the main cotton stems at the seedling stage were about 45 cm high, and the number of leaves was around 13.

**Fig. 1:**
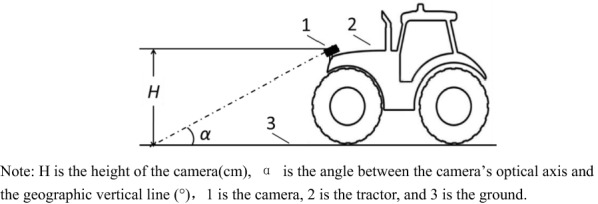
Image acquisition schematic

The camera was mounted in the middle of the front bumper of the JohnDeer 754 tractor, which was directly in front of the cotton row to be identified for acquisition (see the camera installation position in Fig. 1). To avoid the obstruction of the camera by the cotton, the camera was mounted at a height higher than the height of the cotton plant. Specifically, the camera was positioned 100 cm from the ground and the angle between the camera's optical axis and the plumb line was θ = 65°. The frame rate of the captured color video image was 30 frames/s, and the size of each frame was 640 × 480 pixel. At the same time, the developed hardware environment was 3.2 GHz with 16 GB memory, and the software environment was Microsoft Visual C + + 2010. Apart from that, the image processing was developed based on MIAS software from Beijing Modern FuBo Technology inc. under a Windows XP operating system.

### Detection of inter-row lines in broken rows of crops

In this study, the image was converted to grayscale map by using ExG (Excess Green Index), and the cotton rows were segmented using OTSU, which were edge detected by using Canny’s algorithm, respectively.

**Fig. 2:**
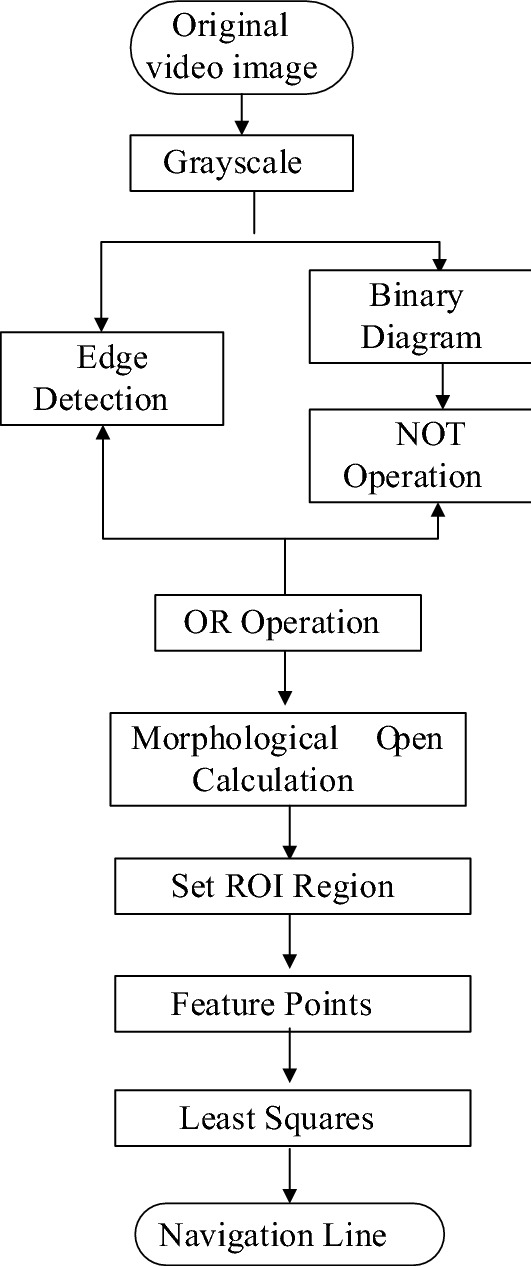
Flow chart of seedling line detection of seedling cotton

The logical NOT operation was used on the result of edge detection, and the new result was compared with the grayscale map by OR Logic Operation. Furthermore, the gap between two cotton rows was found. The central region of the gap was extracted, and then the feature points of the central connection domain were found. Lastly, the least squares method was adopted to fit the inter-row line of the two narrow rows of cotton. Figure 2 shows the main flowchart of the inter-row lines in the broken rows of cotton.

### Image segmentation of cotton rows

#### Edge detection

To reduce row adhesion and segment cotton rows more accurately, this study combined edge detection with binary images to accurately detect the contours of cotton rows. Indeed, the Canny operator could find as many edges in the image as possible [18–20]. Thus, the following edge detection was performed by using the Canny operator on the grayed-out seedling image, to minimize the missed and false detections and pinpoint the centers of two narrow rows of cotton.

#### Binarization and expansion of images

The cotton rows and the background between rows were segmented correctly. Since there was basically no interference from weeds in the cotton field at the seedling stage, a simple and stable segmentation could be performed by OTSU on the green significant grayscale map using a global threshold. At the lower end of the image, due to the viewpoint and the variability of cotton growth, there was a problem of row adhesion in seedling cotton at the upper end of the segmented image. However, since the seedling cotton was at the seedling stage and the leaves were small, the row break caused by the disconnected gaps between branches and leaves within a row of cotton affected the extraction of the connected regions.

To solve the problem of plant out of seedling breakage within a row while ensuring that the two rows of cotton are not stuck together in the process of expansion, a 7 × 21pixel structural element is used to perform five expansion operations on the binary image, so that the cotton rows within the binary image form a connected domain within a row.

Additionally, a 7 × 21pixel structural element was used to perform five expansion operations on the binary image, with the purpose to solve the problem of plant out of seedling breakage within a row, and ensure that the two rows of cotton are not stuck together in the expansion process. In this way, the cotton rows within the binary image could form a connected domain within a row. To accurately extract the cotton row contour, it is necessary to first connect the plant connected domains within a row of cotton.

**Fig. 3:**
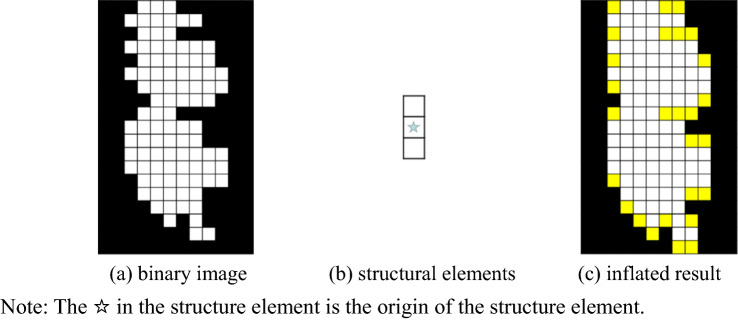
Expansion sketch of binary image

Figure 3 is a schematic diagram of the expansion of binary image. In this case, Fig. 3a shows the binary image calculated by OTSU, with white pixels as the target (cotton rows) and black pixels as the background (soil or mulch between rows). Figure 3b displays the structural element, and the pixel with a pentagram indicates the origin of the structural element. The white pixel is 1, while the black pixel is 0. During the expansion process, each pixel in the binary image is scanned using the structure origin, and the structure element is “summed” with each pixel. If the result is 1, the value of the pixel in the binary image is set to 1, otherwise it is set to 0. Apart from that, the yellow box in Fig. 3c is the pixel whose result is 1 after the expansion. Compared with the white area before the expansion, the white area after the expansion increases in the direction of the vertical axis, but it does not affect the width of the white area on the horizontal axis.

### Inter-seeding line detection

#### Center area selection

The connected domain with area less than 50 pixel was removed, and the distance from centroid of each connected region to the vertical line in the image was calculated. Then the region where the form center had the shortest distance from the vertical line in the image was kept as the center cotton row to extract the navigation line, the purpose of which was to remove the noise interference in the image. Afterwards, the rest of the connected regions were excluded.

**Fig. 4:**
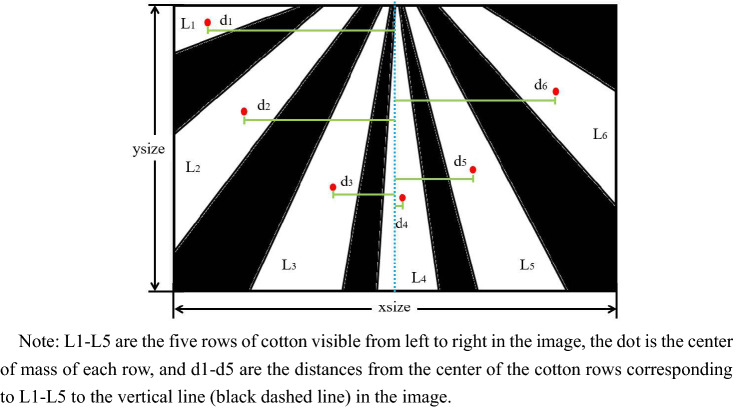
Extraction of central crop rows

The detected edge point pixels of the cotton rows were stored in the array T, and the two rows of cotton closest to the vertical line in the image were found. Besides, the gap between these two rows of cotton was extracted. The cotton rows after OTSU had the adhesion problem, and the adhesion problem of cotton rows was more obvious in the expanded binary image. For the expanded image, the image was inverted by the center connected domain extraction [21–25]. Furthermore, the pixel values of the two rows of cotton edge points in the inverted image were set to 255. The center connected domain acquisition map between rows of cotton in Fig. 4.

**Fig. 5:**
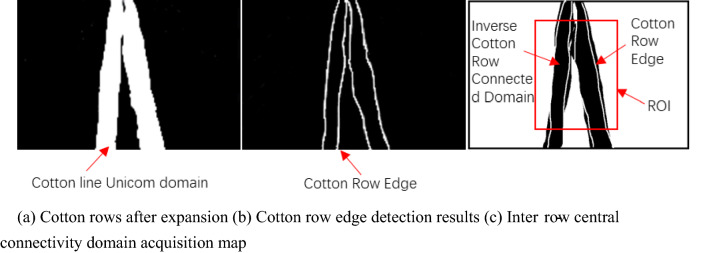
Cotton line edge point detection effect map

The ROI was set for the central connected domain so as to accurately extract the cotton row outline [26, 27]. Furthermore, the horizontal axis of the leftmost pixel of the left connected domain was extended by 50 pixels to the left, while the horizontal axis of the leftmost pixel of the right connected domain was extended by 50 pixels to the right, as the width of the ROI region. Since the seedlings were more likely to break at the lower end of the image, while the seedlings were more likely to stick at the upper end of the image, the height of the ROI region was set to, and all subsequent calculations were limited to the interior of the ROI region Fig. 5.

Since the navigation line should be in the middle of two rows of cotton in a monopoly, the gap between the two rows is the area where the navigation line is located. In the previous step, the middlemost two rows of cotton had been found. The cotton, as the target, were show as white pixel dot, while the background area between the rows was shown as a black pixel dot. In this step, the soil between the middle two rows of cotton (black pixels) should be used as the processing target.

#### Detection of interplant lines

Since the navigation line should be in the middle of two rows of cotton in a monopoly, the gap between the two rows is the area where the navigation line is located. In the previous step, the middlemost two rows of cotton had been found. The cotton, as the target, was a white pixel dot, while the background area between the rows was shown as a black pixel dot. In this step, the soil between the middle two rows of cotton (black pixels) should be used as the processing target, which is calculated as Formula (1).

1 $$B\left( {x,y} \right)\, = \,0,\,A\left( {x,y} \right)\, = \,255\, \cap \,B\left( {x,y} \right)\, = \,255$$

The background (black pixels) with area less than 1000 pixels was inverted to obtain the connected domain in the middle of the two middlemost rows of cotton. All the pixel points of this connected domain were used as the feature points for straight line fitting by the least squares method to get the interplant line.

For the interplant line, the formula for calculating the lateral error of navigation was calculated as formula (2).

2 $$m\, = \,\sqrt {\left( {x_{1} \, - \,x_{0} } \right)^{2} \, + \,\left( {y_{1} \, - \,y_{0} } \right)^{2} } \,$$

where x and y are the horizontal and vertical coordinates of the vehicle each time it is ready to move forward, ${y}_{1}$ and ${x}_{1}$ are the coordinates of the vehicle’s position after traveling a certain distance; ${y}_{0}$ and ${x}_{0}$ are what the professional considers to be the ideal position of the vehicle when it travels to that position.

The body position pose in intelligent vehicle visual navigation is closely related to three factors: vehicle transverse pendulum angular velocity, vehicle center-of-mass velocity, and center-of-mass lateral eccentricity. Assuming that the motion trajectory remains constant and does not change abruptly during the travel of the vehicle along the visual navigation path, the transverse pendulum angular velocity can be expressed by Eq. (3).

3 $$\begin{gathered} \rho = \left. {\frac{{\ddot{y}}}{{\left( {1 + \dot{y}^{2} } \right)^{3/2} }}} \right|_{x = 0} \hfill \\ \dot{\rho } = v\frac{d\rho /dx}{{ds/dx}} \hfill \\ \omega_{p} = v\rho \hfill \\ \end{gathered}$$

where *x*, *y* are the coordinates of the vehicle center of mass, *v* is the velocity of the vehicle center of mass, *s* is the vehicle trajectory, $\rho$ is the road curvature, $\dot{\rho }$ is the rate of change of the road curvature, and ${\omega }_{p}$ is the predicted transverse angular velocity of the vehicle.

## Experiment results

### Analysis of interplant line detection results in cotton rows at seedling stage

**Fig. 6:**
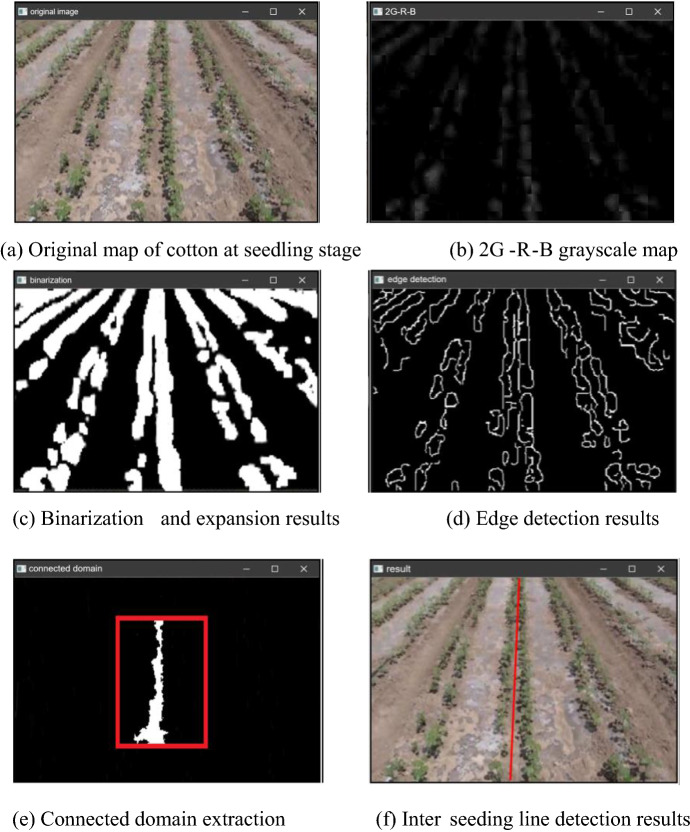
The process of extracting the line between seedlings of cotton

Figure 6 shows the process of acquiring inter-row lines in seedling cotton rows. For seedling cotton planted with one film and six rows, the background between the rows is the mulch and soil, as shown in Fig. 6a, because the cotton plants have small branches, leaves grow sparsely, there is no weed interference, and it is very neat. As the cotton seedlings have just grown out, the leaves are light green and there is a clear gap between the two rows. At the far end of the image (top end), there is row adhesion between the two narrow rows due to the perspective principle. However, at the close end of the image (bottom end), the cotton seedlings appear very sparse and the gap within a row causes a break in the row, and the navigation path detected by the inter-row navigation line is a straight line. Which can represent the trend where the gap between the two narrow rows is located. Despite the obvious film reflection, only the rows of cotton with green leaves are highlighted and the inter-row background (soil and film) is suppressed, as displayed in Fig. 6b.

Due to the principle of perspective, there is a sticky connection between two narrow rows. The more it is against the upper end, the more serious the sticky phenomenon is. However, as the middle two narrow rows of cotton face the camera, with the bottom in the image as the center, the outward direction is the direction of the line of sight, and the middle two narrow rows of cotton shade each other in the same direction as the planting direction of the cotton rows basically, which makes the row break phenomenon not severe. Nevertheless, the planting direction has a certain angle with the direction of the line of sight. Because of the principle of perspective, the more the cotton rows on both sides of the image, the more serious the breakage phenomenon. A rectangular structure with vertical orientation is used for expansion and the row breaks in the image are in a vertical orientation. It can be observed that the row breaks are much less severe and only the obvious missing seedlings can be seen in the expanded binarization. The inverse operation is performed on the binary image of cotton rows after expansion in Fig. 6c, and then calculated with the result of edge detection in Fig. 6d. The outline of cotton rows expands outward during expansion, which strengthens the effect of row adhesion, but its inverse image and the result of edge detection are affected. To find the intersection, the edges cut the expanded cotton rows, and a more accurate cotton row contour is obtained. The two narrow rows are separated by the detected cotton row edges. To further solve the problem of row adhesion and row breakage, the ROI region is set in the middle part of the image to further avoid the impact of row adhesion and row breakage on detection, as shown in the red box in Fig. 6e. Besides, the image within the ROI region is inverted again to obtain the middle two narrow rows of cotton middle gap connected domain. Removing the small area can help remove the excess part of the cut edge, and the trend of the connected domain is the same as the gap between the two narrow rows of cotton. The connected domain obtained in the ROI region is the middle gap between the two narrow rows of cotton, and the results obtained by fitting a straight line to the connected domain using least squares are displayed in Fig. 6f. The final straight line detection results obtained are consistent with the results obtained by human eye observation.

### Adaptation analysis of inter-seed line detection algorithm

**Fig. 7:**
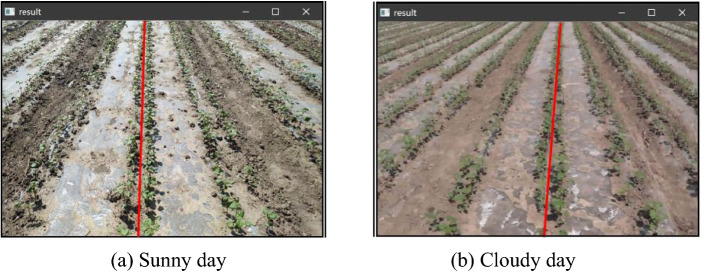
Results of inter-row line detection in cotton at seedling stage under different weather conditions

This paper not only conducted an inter-row line detection study on cotton planted in wide and narrow rows of six rows of one film and at the seedling stage in a row-break crop, but also detected the inter-row lines of cotton at the seedling stage on sunny and cloudy days. The results have been shown in Fig. 7, in which Fig. 7a, b present the results of inter-row navigation line detection for seedling cotton on sunny and cloudy days, respectively.

**Fig. 8:**
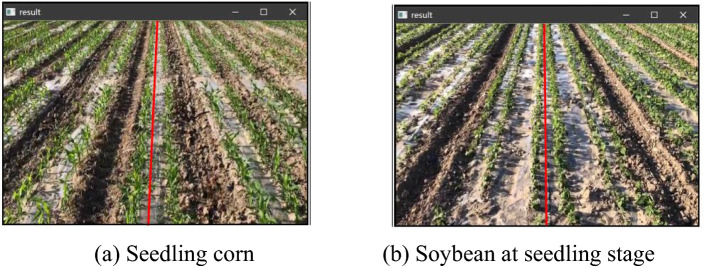
Test results of lines between seedlings of different crops

We carried out a seedling inter-row line detection study on corn and soybean that were also sown in double rows, as shown in Fig. 8. The navigation line detection was performed for seedling corn and seedling soybean crops at the seedling stage, to test the suitability of the algorithm. Figure 8a displays the results of navigation line detection for seedling corn, while Fig. 8b presents the results of navigation line detection for seedling soybean. Since the leaves of seedling corn are narrow and long, the Canny operator with Gaussian radius of 0.5 is used to reduce the excessive edge lines caused by the excessive length of the leaves, and the edges of corn rows obtained by this operator are less. However, the soybean leaves are ovate, like the palm-shaped leaves of cotton, and the Canny operator with Gaussian radius of 0.3 was used for edge detection.

### Error analysis

In this study, inter-row navigation line detection was performed for seedling cotton, seedling corn, and seedling soybean, respectively. Beyond that, three video segments were obtained for each of the three crops in the seedling state, as displayed in Table 1.

**Table 1:** Test results of lines between seedlings of different crops

Video ID	Total frames	Start frame	Consecutive frames	Accuracy rate (%)	Cause of the error
Cotton 1 (Sunny day)	876	/	/	100	/
Cotton 2 (Sunny day)	832	387/871	8/4	98.5	Missing seedlings/rut marks
Cotton 3 (Cloudy day)	651	732	8	98.7	Missing seedlings
Corn 1 (Sunny day)	976	613	16	98.3	Missing seedlings
Corn 2 (Sunny day)	832	347	11/9	97.5	Missing seedlings
Corn 3 (Cloudy day)	995	553	7/10	98.2	Missing seedlings/rut marks
Soybean 1 (Sunny day)	814	285	14	98.2	Missing seedlings
Soybean 2 (Sunny day)	665	339	9	98.6	Missing seedlings
Soybean 3 (Cloudy day)	512	479	8	98.4	Rut marks

The algorithm was verified through multiple video images collected. The detection results were correct, according to the detection results observed from the judgment of experienced personnel. The inter-row navigation line detection for seedling crops is the gap between two narrow rows of crops, while the variability of seedling crops in the early and late emergence causes the randomness of missing seedlings [28–30]. Besides, the algorithm is sensitive to the lack of seedlings. Thus, the lack of seedlings significantly affects the results of navigation line detection.

The impact of rutted seedlings on the results of navigation line detection has been divided into two cases. If the rutted seedlings are in the ROI area, the seedlings of the crops pressed by the rutted seedlings are missing, which significantly affects the navigation line. If the rutted seedlings are outside the ROI area, the results of navigation line detection will not be affected. The main influence on the detection results is the lack of seedlings and rutting marks.

The accuracy of the line of sight for seedling cotton was 99.2%, corresponding to a following accuracy of ± 1 cm. The average processing time was 6.63 ms per frame. Besides, 98.1% of the line of sight for seedling corn was accurate, with an average processing time of 6.97 ms per frame. In addition, 98.4% of the line of sight for seedling soybean was accurate, with an average processing time of 6.72 ms per frame. In addition, the standard deviation of lateral deviation is 2 cm, and the standard deviation of heading deviation is 0.57 deg.

Missing seedlings accounted for the majority of cases among the different error detection scenarios. Among the three different crops, the accuracy of seedling detection was higher for cotton than for the other two crops, because cotton is planted in a film-laying manner and the transparent film avoids the clutter of the background between the rows when covering the soil between the rows and prevents the growth of weeds between the rows. For the cotton in the seedling stage, the color in the image was more obvious on sunny days, so that the detection was better.

## Discussions

### Gray-scale map analysis of seedling cotton application images in RGB color space

**Fig. 9:**
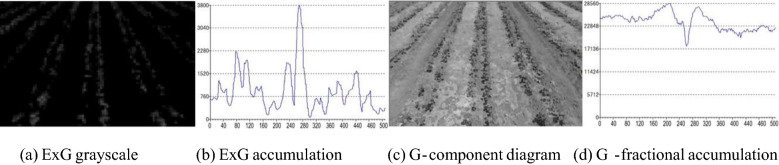
Gray-scale image contrast of cotton in seedling stage

Figure 9 shows the comparison of grayscale maps of cotton at the seedling stage. Among them, Fig. 9a displays the ExG grayscale map, Fig. 9b presents the results of pixel accumulation in the vertical direction to the ExG grayscale map. Besides, Fig. 9c shows the G component grayscale map, and Fig. 9d displays the results of pixel accumulation in the vertical direction to the G component grayscale map.

Comparing Fig. 9a, b, it can be observed that the G component is enhanced in the ExG grayscale map, and the cotton rows can be clearly distinguished. The pixel accumulation results in the ExG grayscale map contain richer pixel change information, and the peaks formed by the pixel accumulation values are set by the cotton rows. Comparing Fig. 9(c, d), it can be seen that although the G component of cotton leaves is higher, the soil color is lighter and each RGB color component of the soil is higher. Thus, although the cotton rows can also be clearly seen in the G component grayscale map, the effect is not as obvious as that seen in the ExG grayscale map, and the fluctuation pattern of the accumulated values corresponding to the cotton rows cannot be identified in its pixel accumulation results.

### Effect of different edge operators on cotton row extraction

**Fig. 10:**
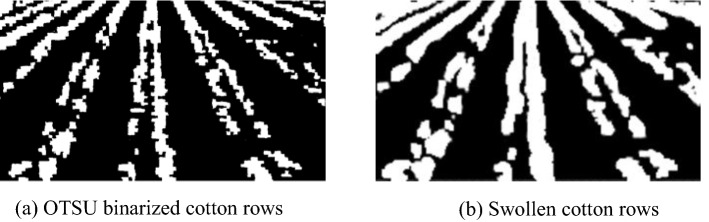
Cotton row binary map comparison

Figure 10 shows the binary image of seedling cotton and the effect after expansion, where Fig. 10a displays the result of automatic threshold segmentation using OTSU, and seedling cotton at the seedling stage has row adhesion as well as row breakage. Figure 10b shows the binary image with expansion of the OTSU segmentation result. Beyond that, there is less row breakage in this image, while the row adhesion is more obvious than in Fig. 10a.

**Fig. 11:**
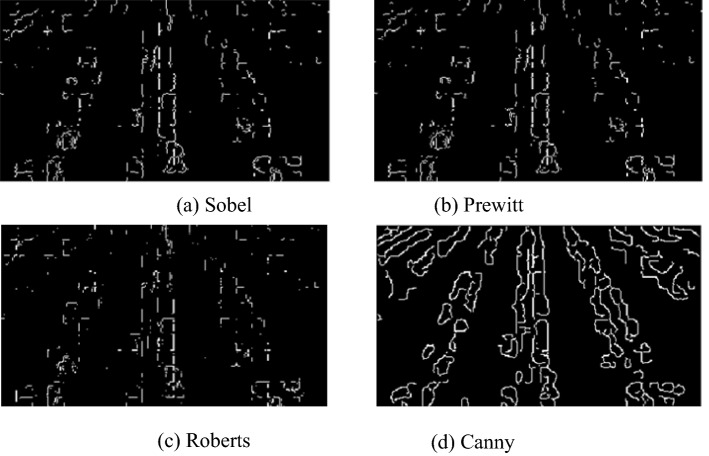
Comparison of detection results of edge detection operators

Figure 11 shows the results of cotton row edge detection in the ExG grayscale map, which compares the effect of detection by different edge detection operators. Figure 11a–d presents the detection results using Sobel operator, Prewitt operator, Roberts operator and Canny algorithm, respectively. Indeed, the canny algorithm adopts the second-order differentiation for edge detection. Comparing the other three results of edge detection using the first-order differentiation, it can be observed that the obtained cotton row edges are more complete.

**Fig. 12:**
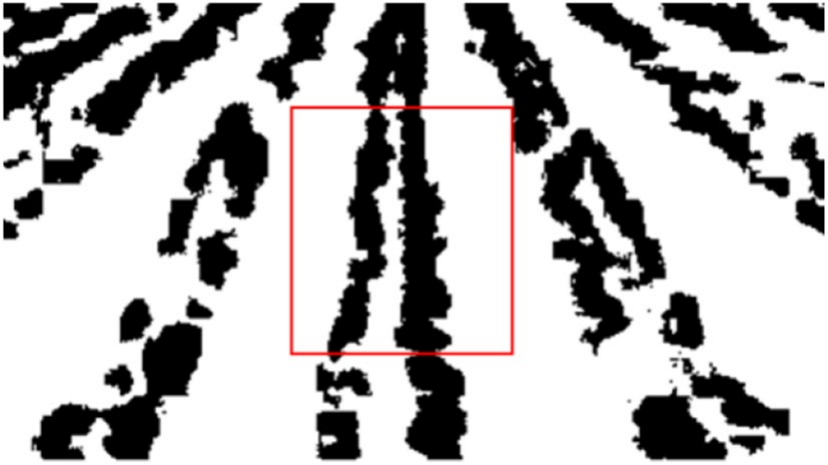
Test results of navigation line of cotton seedling

Since the detection results of the Canny operator outperform the other detection operators, the detection results of different Gaussian radii are compared again in the Canny operator with Gaussian radii of 0.04, 0.1, 0.3 and 0.5, respectively. For the Gaussian radius of 0.04 and 0.1, the cotton row edge in the detection result is too fine, while for the detection result of Gaussian radius of 0.5, the edge information is too little to meet the condition of subsequent removal of row adhesion. Hence, the edge detection of Gaussian radius of 0.3 has been chosen. With Gaussian radius of 0.3, the result is presented in Fig. 12, in which the red box is the ROI region, and the center of mass of each connected domain is calculated in this region. Apart from that, the connected domain in which the center of mass is nearest to the vertical line in the image is selected, and all the pixel points in this connected domain are used as feature points. What’s more, the least squares method is adopted to fit the navigation line of cotton at the seedling stage.

## Conclusions

For cotton grown in one film with six wide and narrow rows of machine harvested cotton, this paper proposed a method for cotton navigation path detection during the seedling stage. The seedling leaves of seedling cotton are small, and there are some gaps between two narrow rows. Apart from that, row adhesion and row breakage increase the complexity of visual navigation path detection. For these two disturbances, this paper proposed a detection algorithm for seedling column lines.

In a film six wide and one narrow planting method (machine picked cotton), the seedling leaves are small, and there are some gaps between the two narrow rows. Meanwhile, the near row breakage and distant sticking phenomena all cause the great interference to the extraction accuracy of cotton rows. To overcome these disturbances, this paper developed algorithms for a film of six rows of seedling cotton through wide and narrow row planting methods. On the one hand, using the rectangular structure for extension of the binary image can effectively solve the row breakage problem. On the other hand, using the extended binary map and the cotton row edge results for calculation of the ExG grey map respectively can effectively solve the row sticking problem. At the same time, the inter-row line of two narrow rows of cotton at the seedling stage was obtained accurately and reliably by finding the connecting domain between two narrow rows of cotton in the middle of the image through morphological operations and fitting a straight line. Besides, the video inspection was performed on corn and soybean at the seedling stage, so as to verify the generalization ability of the algorithm.

As shown by the experimental results, the line-of-sight accuracy for seedling cotton was 99.2% with an average processing time of 6.63 ms per frame. In addition, the line of sight accuracy for maize seedlings was 98.1%, with an average processing time of 6.97 ms per frame. Soybean seedlings were 98.4% accurate for line of sight, with an average processing time of 6.72 ms per frame. Moreover, the accuracy of the navigation line detection was no less than 98%. There are differences in leaf shape and sowing method of different seedling crops, so the navigation results also differ. The average results of nine field trials conducted on cotton, corn and soybeans showed that, the standard deviation of lateral deviation is 2 cm, and the standard deviation of heading deviation is 0.57 deg.

As the cotton at the seedling stage was detected differently from cotton at the bud and boll stage navigation lines, seedling cotton had been detected by fitting a straight line to the gap between two rows of cotton. However, for cotton at the bud and boll stage, the detection was done on the rows of cotton. Particularly, the navigation line was determined when the crop row was bent at the seedling stage, because the sowing line is not straight.

## Data Availability

The primary images that were acquired from cotton fields and the extracted features datasets used and/or analyzed during the current study are available from the corresponding author on reasonable request. All the other data generated or analyzed during this study are included within this article.
